# PSYCHOSOCIAL CHARACTERISTICS AND DEPRESSIVE SYMPTOMATOLOGY AMONG MEXICAN OLDER ADULTS

**DOI:** 10.1093/geroni/igz038.3190

**Published:** 2019-11-08

**Authors:** L Paige B Downer, Rebeca Wong

**Affiliations:** The University of Texas Medical Branch, Galveston, Texas, United States

## Abstract

The incidence of mental health problems, specifically depression, are increasing among older adults. Previous research identified psychosocial characteristics related to mental resiliency, such as increased conscientiousness and internal locus of control, as contributing to improved health behaviors and better physical and mental health outcomes. This study identifies the association between these psychosocial traits and depressive symptomatology among Mexican adults aged 50 and older using Wave 4 of the Mexican Health and Aging Study (2015). We hypothesize that psychosocially ‘strong’ older Mexican adults, those with higher levels of conscientiousness and an internal locus of control, will report fewer depressive symptoms; we theorize that gender moderates this relationship. Older Mexican adults’ mental health status was measured through depressive symptomatology using a 9-item version of the Centers for Epidemiologic Studies-Depression (CES-D) scale. Conscientiousness was measured using a 6-item sub-dimension of the “Big 5” personality scale. Locus of control was measured using an 8-item scale adapted from Rotter (1966). Multivariable linear regression was performed and adjusted for socio-demographic characteristics and comorbid chronic health conditions. Depressive symptoms were lower with higher age and formal education; however, older adults in rural environments who are divorced/separated or widowed exhibit higher depressive symptoms. The association between conscientiousness and depressive symptoms differed by gender. Locus of control and conscientiousness influenced depressive symptomatology in men, however, only conscientiousness impacted women’s depressive symptoms. Continued research is needed to assess psychosocial changes over the life-course as they contribute to resilience from adverse life-events that influence mental health in old age.

